# Publisher Correction: ERK-mediated phosphorylation regulates SOX10 sumoylation and targets expression in mutant BRAF melanoma

**DOI:** 10.1038/s41467-018-03710-1

**Published:** 2018-04-06

**Authors:** Shujun Han, Yibo Ren, Wangxiao He, Huadong Liu, Zhe Zhi, Xinliang Zhu, Tielin Yang, Yu Rong, Bohan Ma, Timothy J. Purwin, Zhenlin Ouyang, Caixia Li, Xun Wang, Xueqiang Wang, Huizi Yang, Yan Zheng, Andrew E. Aplin, Jiankang Liu, Yongping Shao

**Affiliations:** 1 Frontier Institute of Science and Technology, and Key Laboratory of Biomedical Information Engineering of Ministry of Education, School of Life Science and Technology, Xi’an Jiaotong University, 710049 Xi’an, China; 2 Department of Cancer Biology, Thomas Jefferson University, 19107 Philadelphia, PA USA; 3 Department of Dermatology, the Second Affiliated Hospital, School of Medicine, Xi’an Jiaotong University, 710004 Xi’an, China; 4 Sidney Kimmel Cancer Center, Thomas Jefferson University, 19107 Philadelphia, PA USA; 5 National & Local Joint Engineering Research Center of Biodiagnosis and Biotherapy, The Second Affiliated Hospital, Xi’an Jiaotong University, 710004 Xi’an, China; 6 Tianjin Key Laboratory of Exercise Physiology and Sports Medicine, Tianjin University of Sport, Tianjin, China

Correction to: *Nature Communications* 10.1038/s41467-017-02354-x, published online: 02 Jan 2018.

In the original version of this Article, financial support was not fully acknowledged. The PDF and HTML versions of the Article have now been corrected to include the following:

The National Basic Research Program (2015CB553602 to J.L.), the National Natural Science Foundation of China (31570777, 91649106, 31770917 to J.L.) and Tianjin Applied Basic and Frontier Tech Major Project (12JCZDJC34400 to J.L.) and Tianjin Higher Education Sci-Tech Development Project (20112D05 to J.L.).

