# Supplementary material for: ERK-mediated phosphorylation regulates SOX10 sumoylation and targets expression in mutant BRAF melanoma
Source: Nat Commun. 2018 Jan 2;9:28. doi: 10.1038/s41467-017-02354-x (PMC5750221; doi:10.1038/s41467-017-02354-x)
Supplement: Supplementary file 1 — Supplementary Information(PDF 3006 kb) [file 41467_2017_2354_MOESM1_ESM.pdf]

Supplementary table 1. Spearman correlation between SOX10 and its target genes based on TCGA and Talantov datasets

TCGA dataset	All (N=473)		BRAF V600 mutation (N=159)	
Gene	Sr	p Value	Sr	p Value
FOXD3	0.137	0.003 **	0.165	0.037 *
MITF	0.260	9.66E-09 ***	0.220	0.005 **
DCT	0.254	2.05E-08 ***	0.177	0.026 **
TYR	0.388	1.84E-18 ***	0.388	4.49E-07 ***

Talantov dataset	All (N=87)	
Gene	Sr	p Value
FoxD3	0.504	6.40E-07 ***
MITF	0.554	2.53E-08 ***
DCT	0.410	7.95E-05 ***
TYR	0.958	6.00E-48 ***

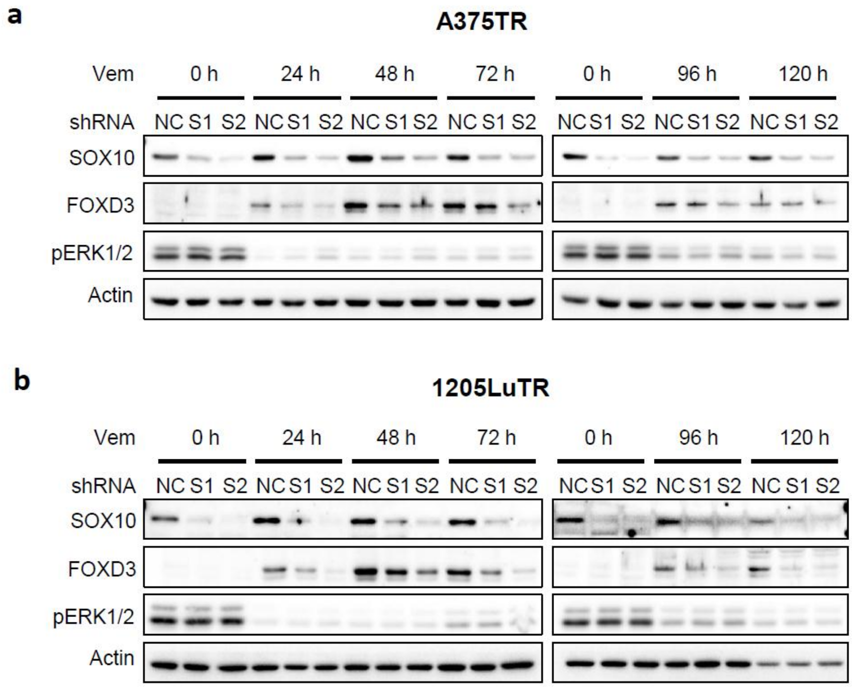

Supplementary Fig. 1. FOXD3 induction in durable in RAFi-treated melanoma cells. A375-TR SOX10 shRNA, 1205Lu-TR SOX10 shRNA and their control cell lines were cultured in the presence of 100 ng/mL Doxycycline for 72 hours, and then treated by 2 μM Vemurafenib for 0-120 hours. Cells were lysed and western blot analyses were performed on indicated proteins. Uncropped images are shown in Supplementary Fig. 16.

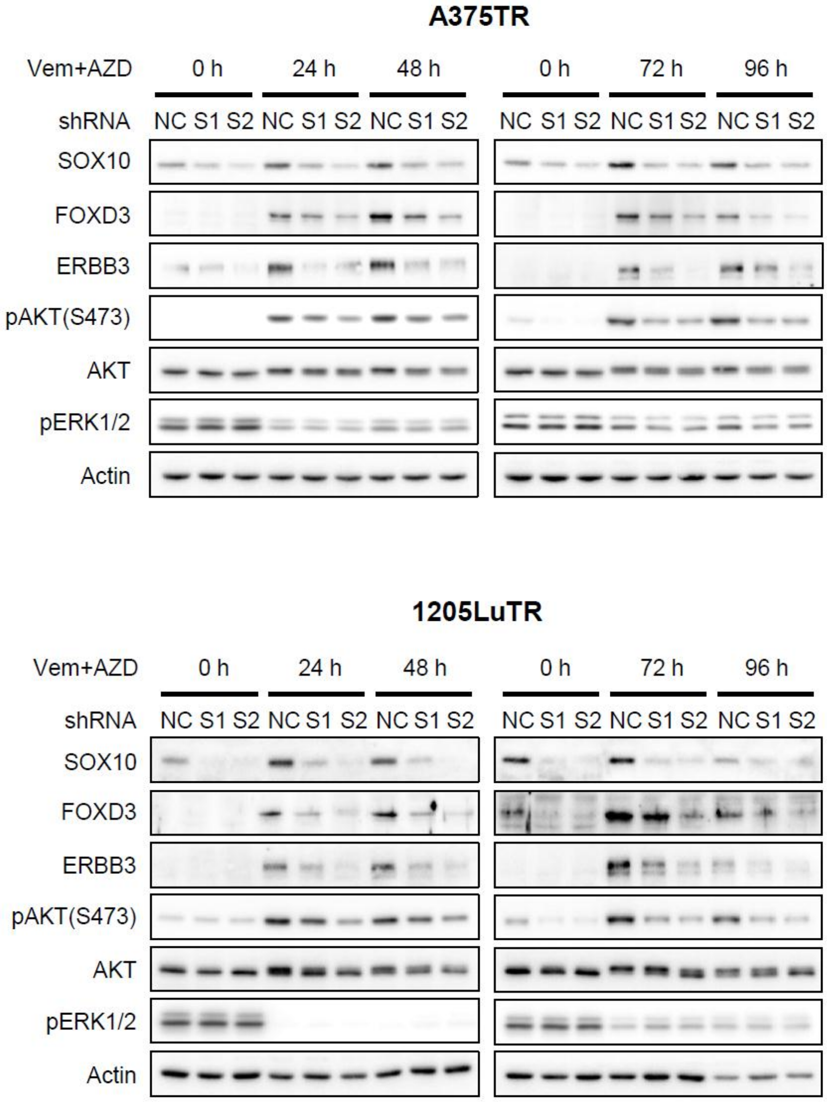

Supplementary Fig. 2. The ERK-SOX10-FOXD3-ERBB3 axis in RAFi/MEKi combo-treated mutant BRAF melanoma cells. A375-TR SOX10 shRNA, 1205Lu-TR SOX10 shRNA and their control cell lines were cultured in the presence of 100 ng/mL Doxycycline for 72 hours, and then treated by 2 μM Vemurafenib plus 5μM AZD6244 for 0-96 hours. Cells were stimulated with with 10 ng/ml NRG1 for 1 hour and lysed for western blot analysis. Uncropped images are shown in Supplementary Fig. 17-18.

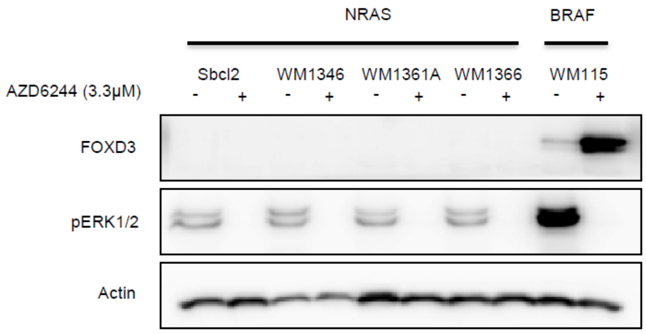

Supplementary Fig. 3. Mutant-NRAS melanoma cells don't express FOXD3. Mutant-NRAS (Sbcl2, WM1346, WM1361A and WM1365) or mutant-BRAF (WM115) melanoma cell lines were treated with or without 3.3μM AZD6244 for 24 hours and lysed for western blot analysis on FOXD3 and phospho-ERK1/2. Actin was used as the loading control. Uncropped images are shown in Supplementary Fig. 19.

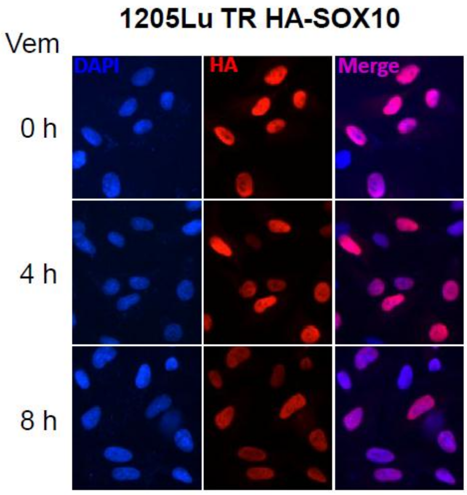

Supplementary Fig. 4. The nuclear localization of SOX10 is not affected by Vemurafenib treatment. 1205Lu-TR HA-SOX10 cells were cultured in the presence of 100 ng/mL Doxycycline for 72 hours, and then treated by 2 μM Vemurafenib for 0, 4, or 8 hours. Cells were harvested for immunofluorescence assay. DAPI was in blue, HA (SOX10) was in red.

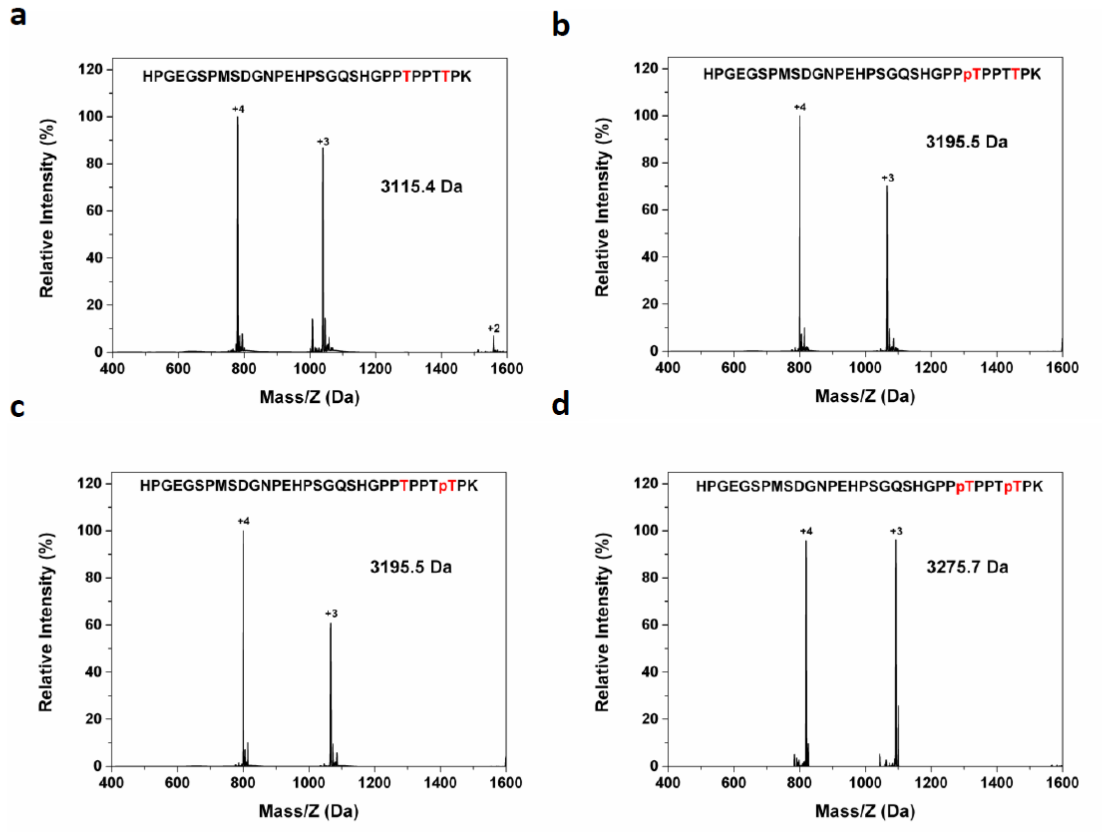

Supplementary Fig. 5. Mass spectrometry results for synthetic SOX10 peptide standards (HPGEGSPMSDGNPEHPSGQSHGPPTPPTTPK). (**a**) non-phosphorylated Sox10 peptide. (**b**) T240-phosphorylated SOX10 peptide. (**c**) T244-phosphorylated SOX10 peptide. (**d**) T240 and T244 double phosphorylated SOX10 peptide. The molecular weight of each peptide was shown.

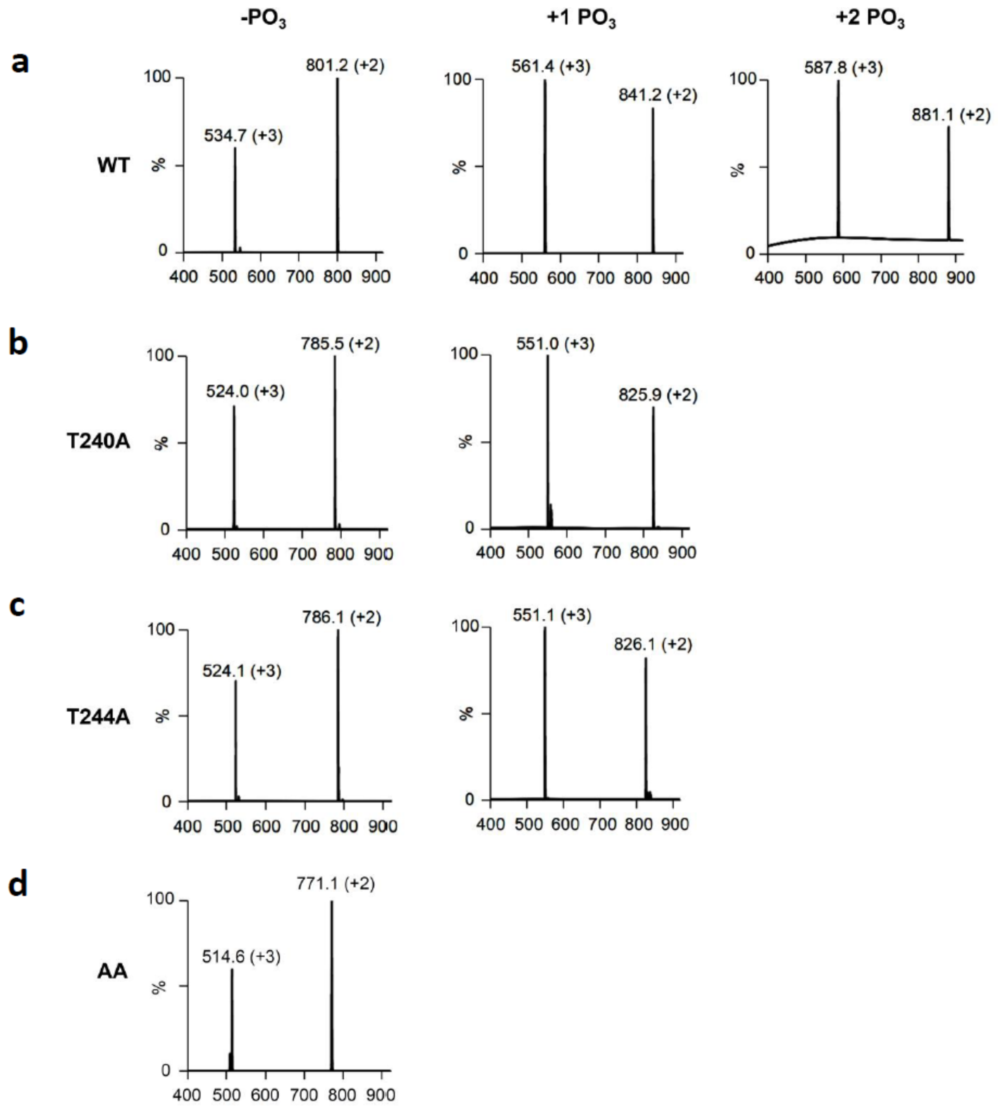

Supplementary Fig. 6. Mass Spectrometry results for reaction products from the ERK2/SOX10 *In vitro* kinase assay. (**a**) Three peptide species corresponding to non-phosphorylated, single-phosphorylated and double-phosphorylated peptides respectively were detected with WT SOX10. Single-, but not double-phosphorylated peptide species were detected with T240A (**b**) and T244A (**c**) SOX10. (**d**) No phosphorylation was detected with AA-SOX10.

a

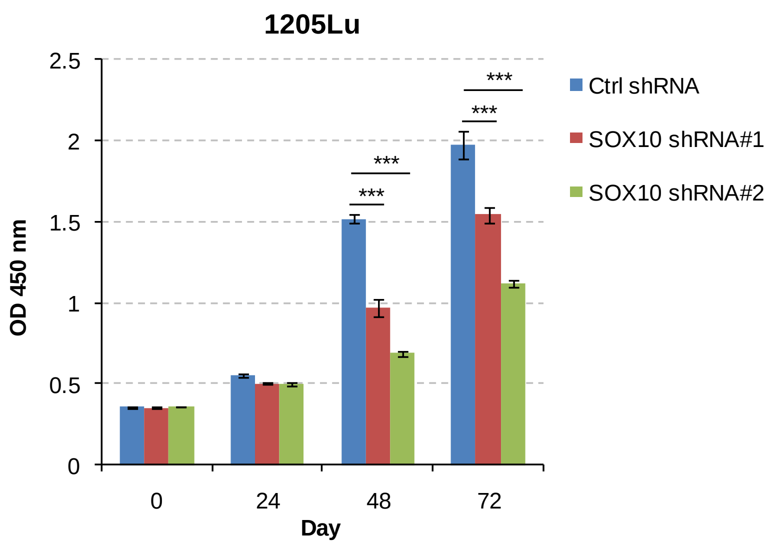

b

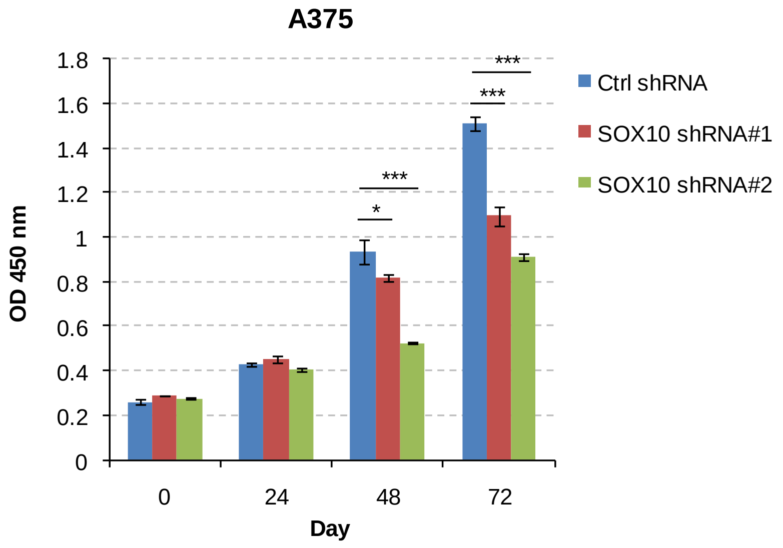

Supplementary Fig. 7. MTT assays on melanoma cells depleted of SOX10. (**a**) 1205Lu-TR Ctrl-shRNA, SOX10 shRNA #1 and #2 cells were treated with 100 ng/ml doxycycline for 72 hours. Cells were then replated at a density of 5000/well in 96 well plates and cell growth was monitored over 72 hours using the MTT assay. Error bars represent standard deviations (N=3), significance was determined by ANOVA one-way test, *, p<0.01; ***, p<0.001. (**b**) Same as (a) except that A375 cells were used.

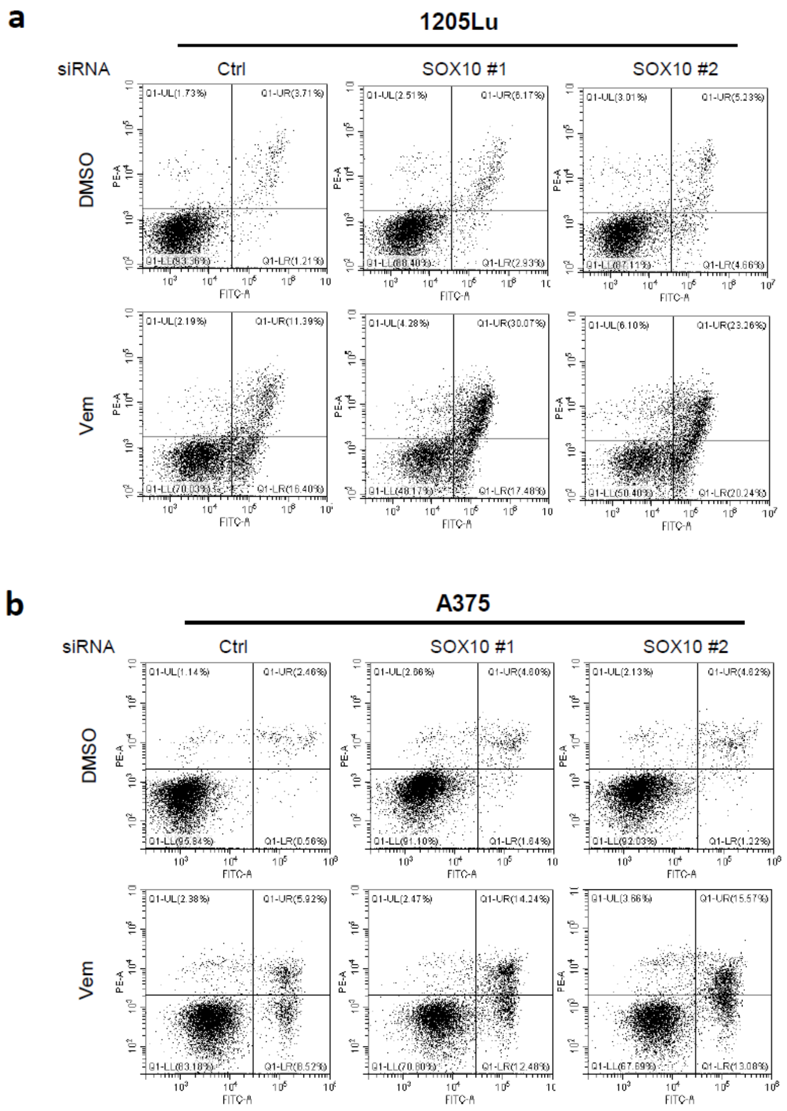

Supplementary Fig. 8. AnnexinV-PI staining of melanoma cells depleted of SOX10. (**a**) 1205Lu were transfected with Control, SOX10#1 or #2 siRNAs for 48 hours and treated with -/+ 10 μM Vemurafenib for additional 48 hours. Cells were then harvested and stained with Annexin-V/PI for flow cytometry analysis. Representative flow traces were shown. (**b**) Same as (a) except that A375 cells were used and cells were treated with 5 μM Vemurafenib for 72 hours.

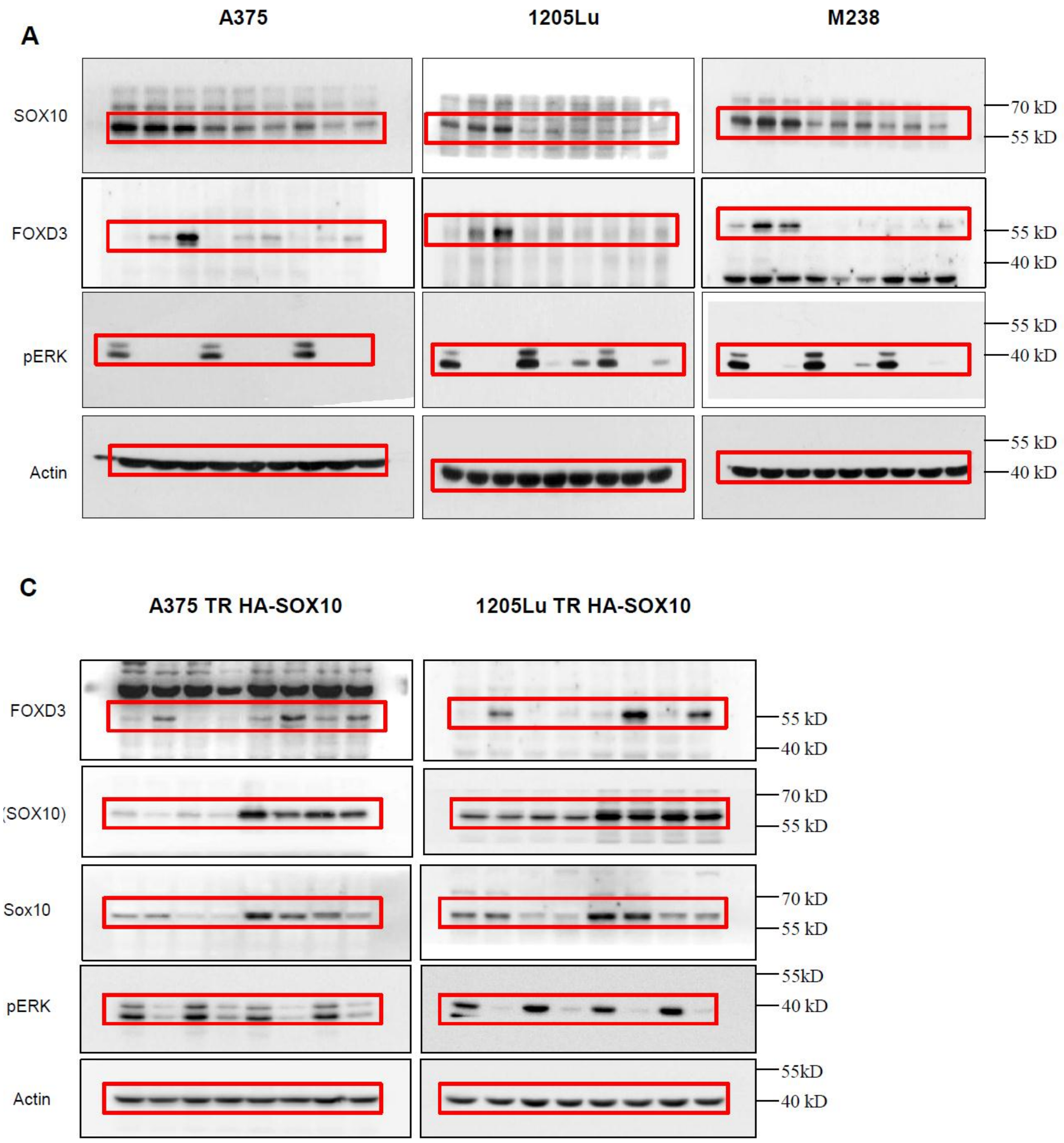

Supplementary Fig. 9. Uncropped western blot images of Fig. 1a and 1c.

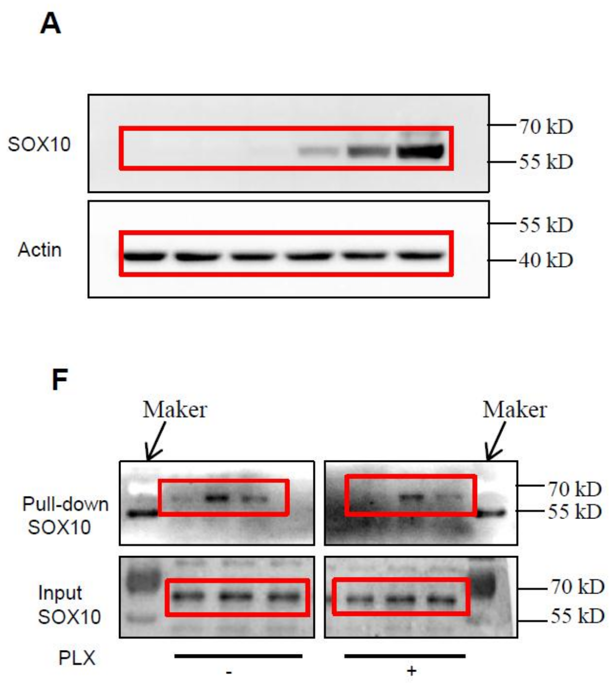

Supplementary Fig. 10. Uncropped western blot images of Fig. 2a and 2f.

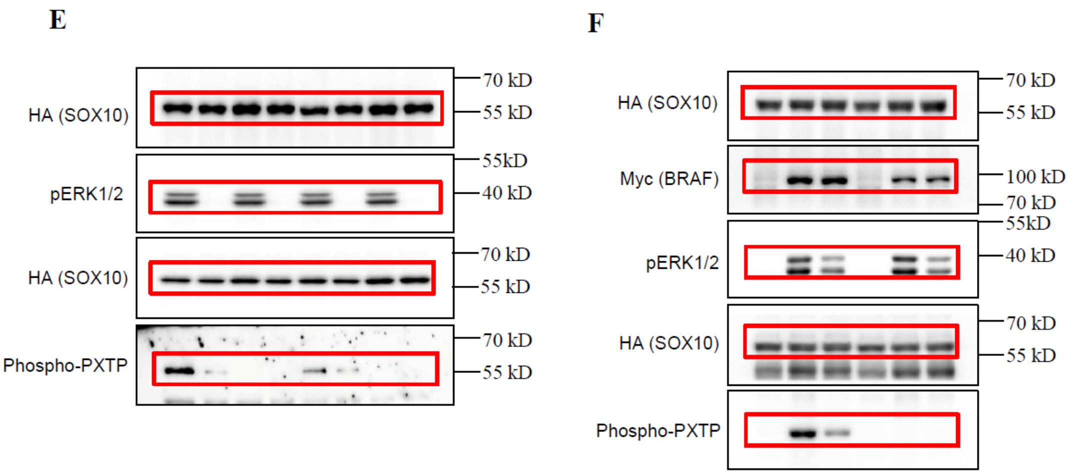

Supplementary Fig. 11. Uncropped western blot images of Fig. 3e and 3f.

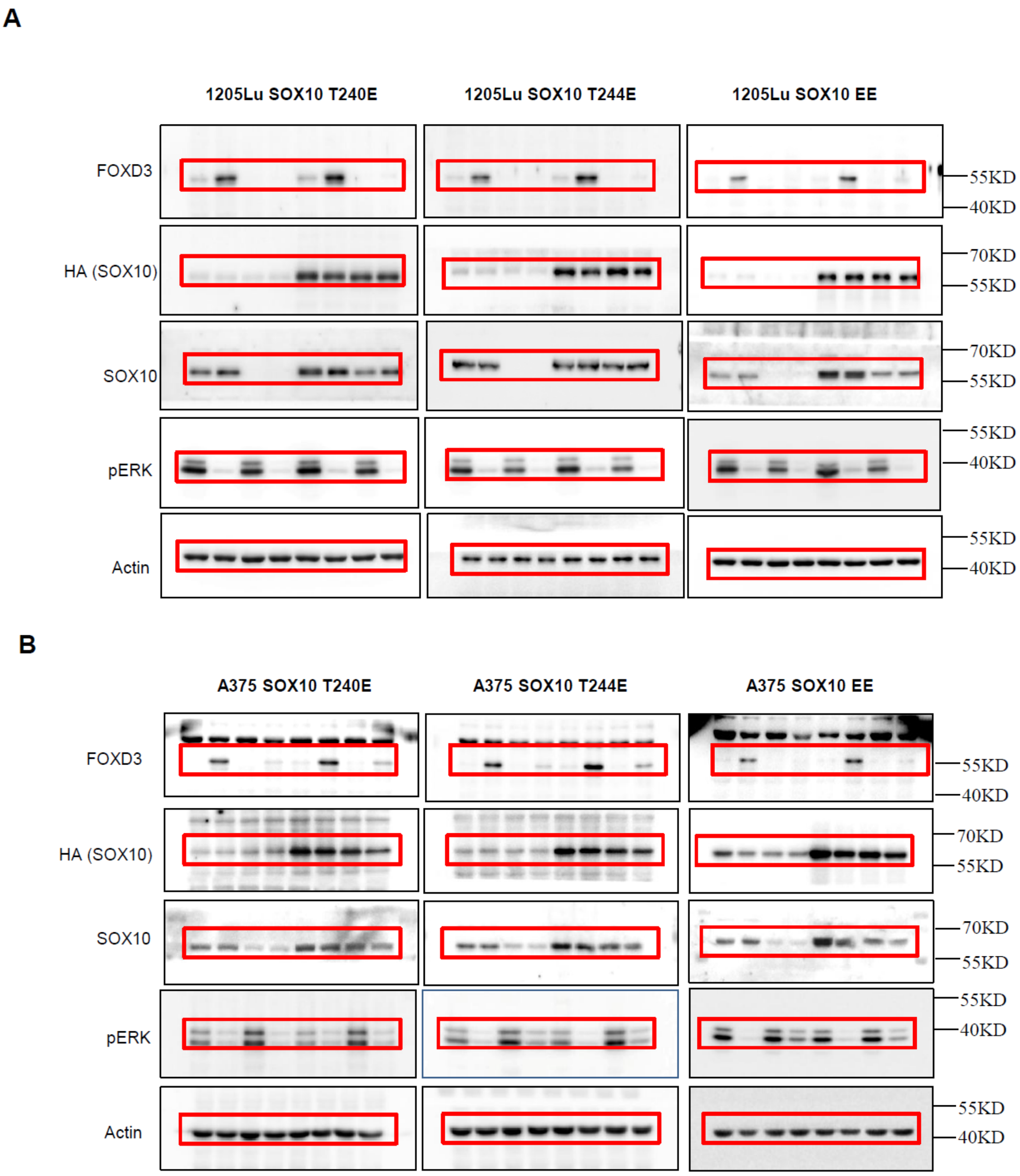

Supplementary Fig. 12. Uncropped western blot images of Fig. 4a and 4b.

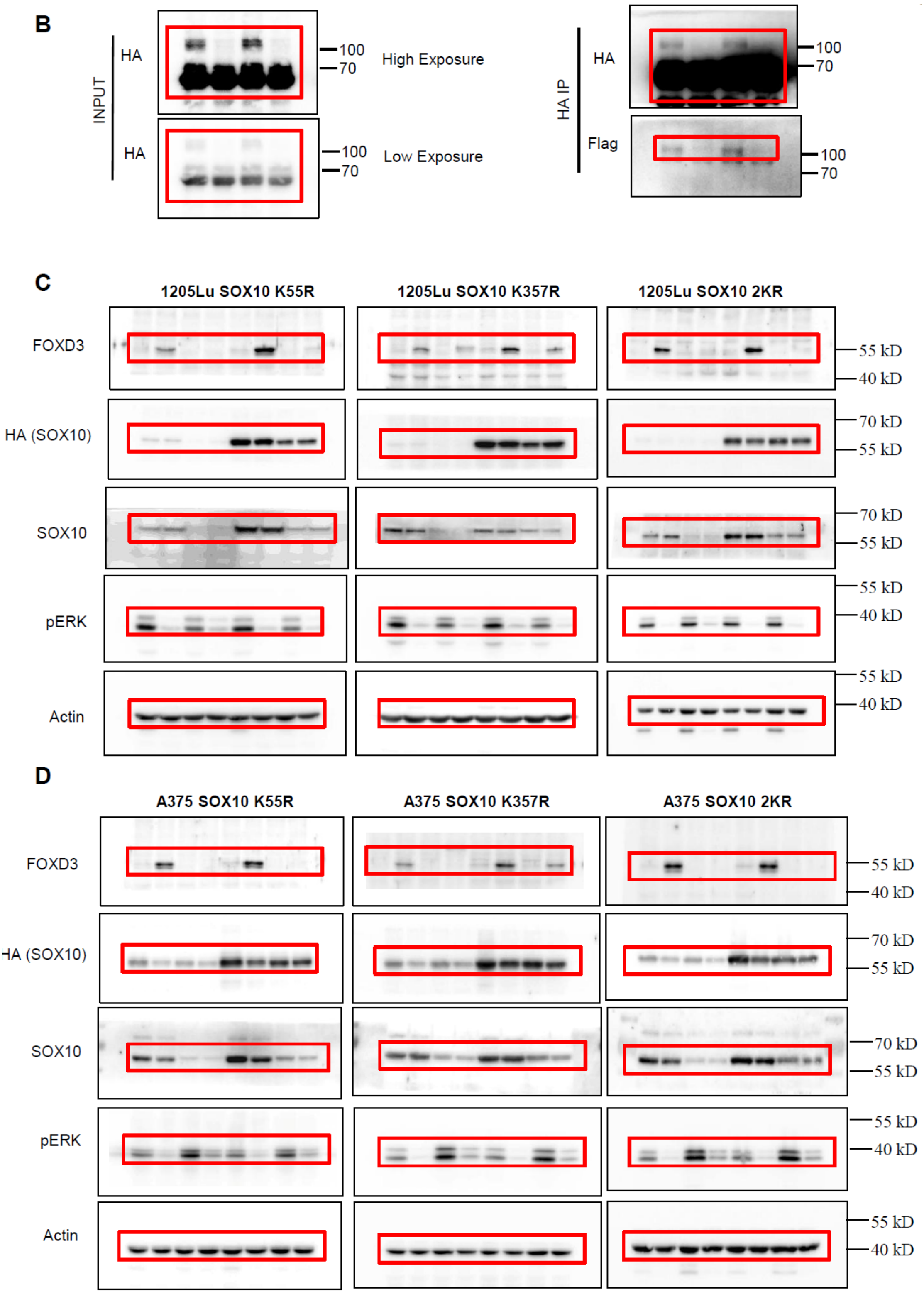

Supplementary Fig. 13. Uncropped western blot images of Fig. 5b, 5c and 5d.

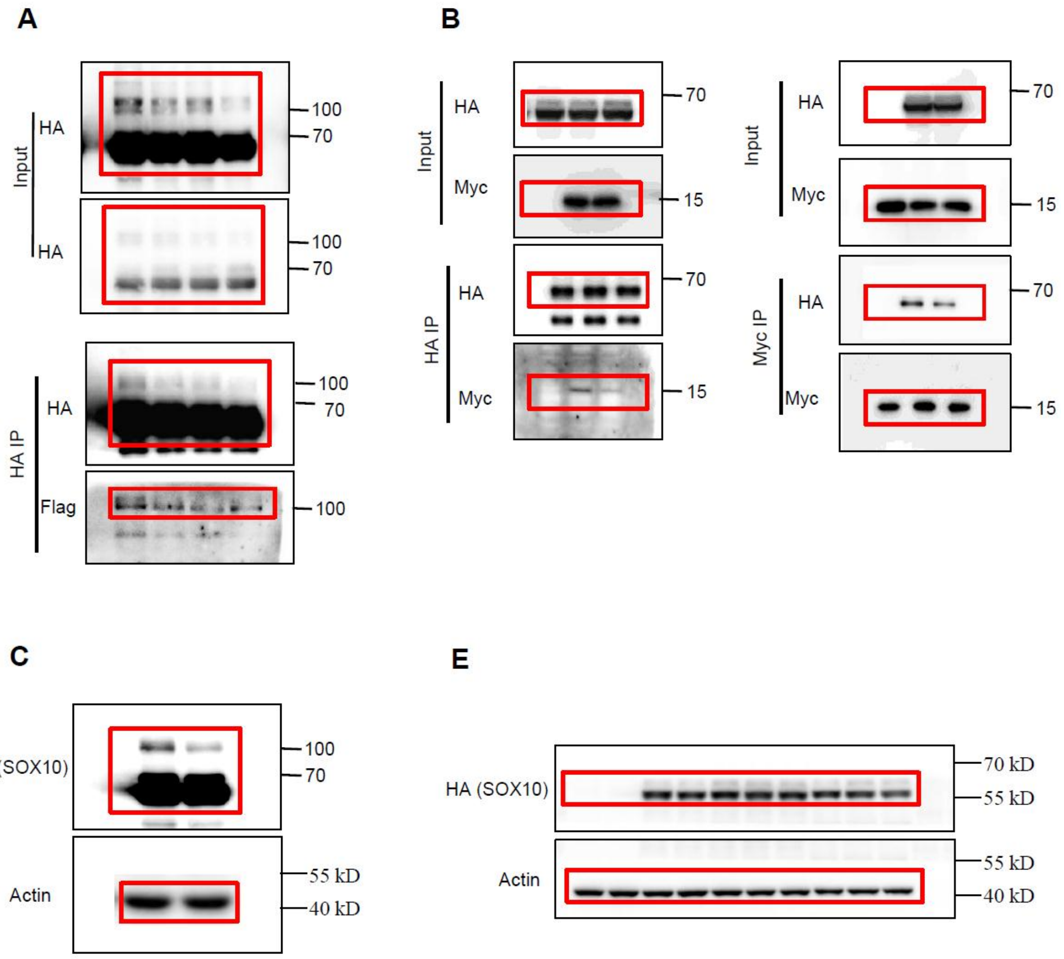

Supplementary Fig. 14. Uncropped western blot images of Fig. 6a, 6b, 6c and 6e.

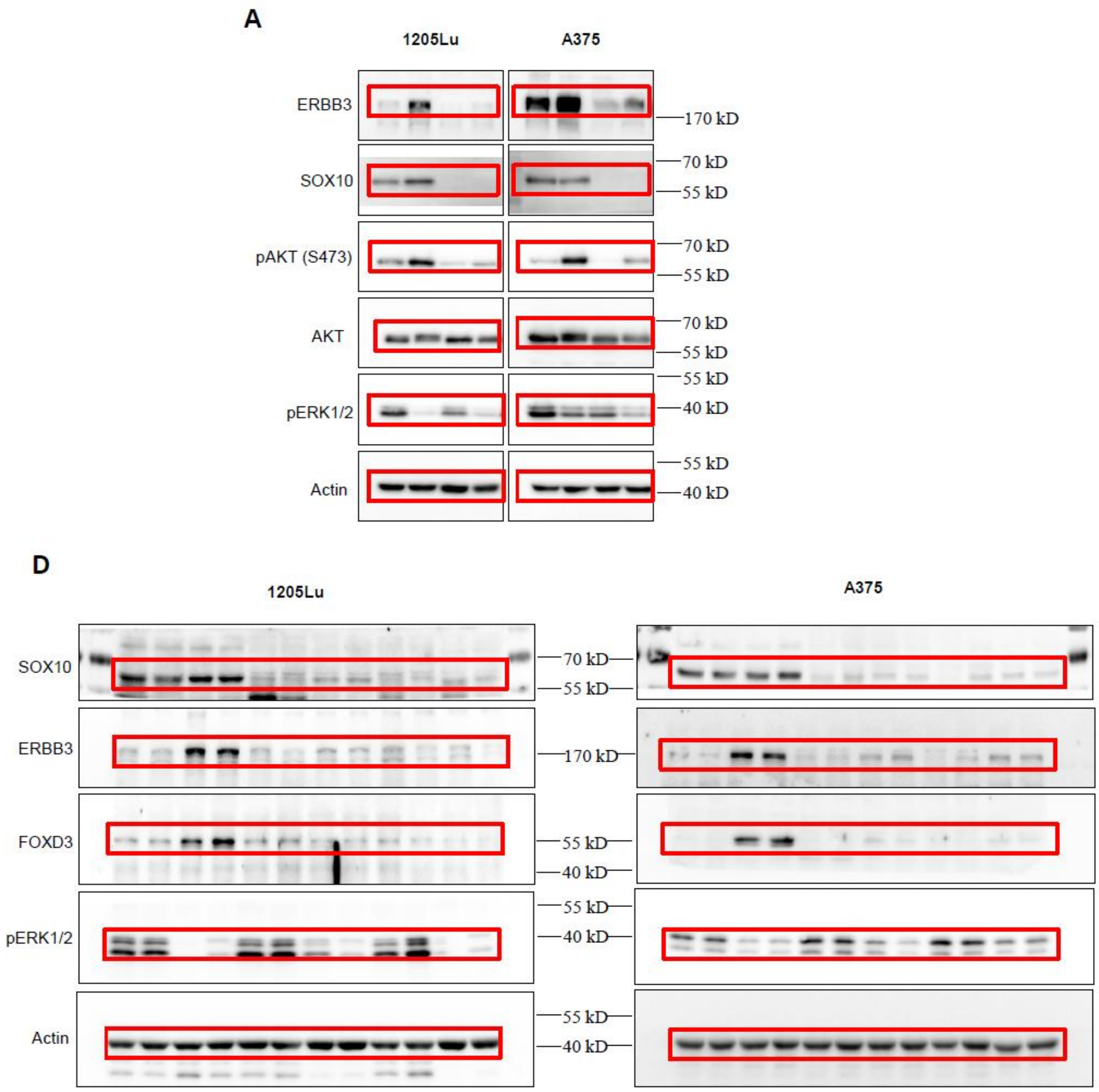

Supplementary Fig. 15. Uncropped western blot images of Fig. 7a and 7d.

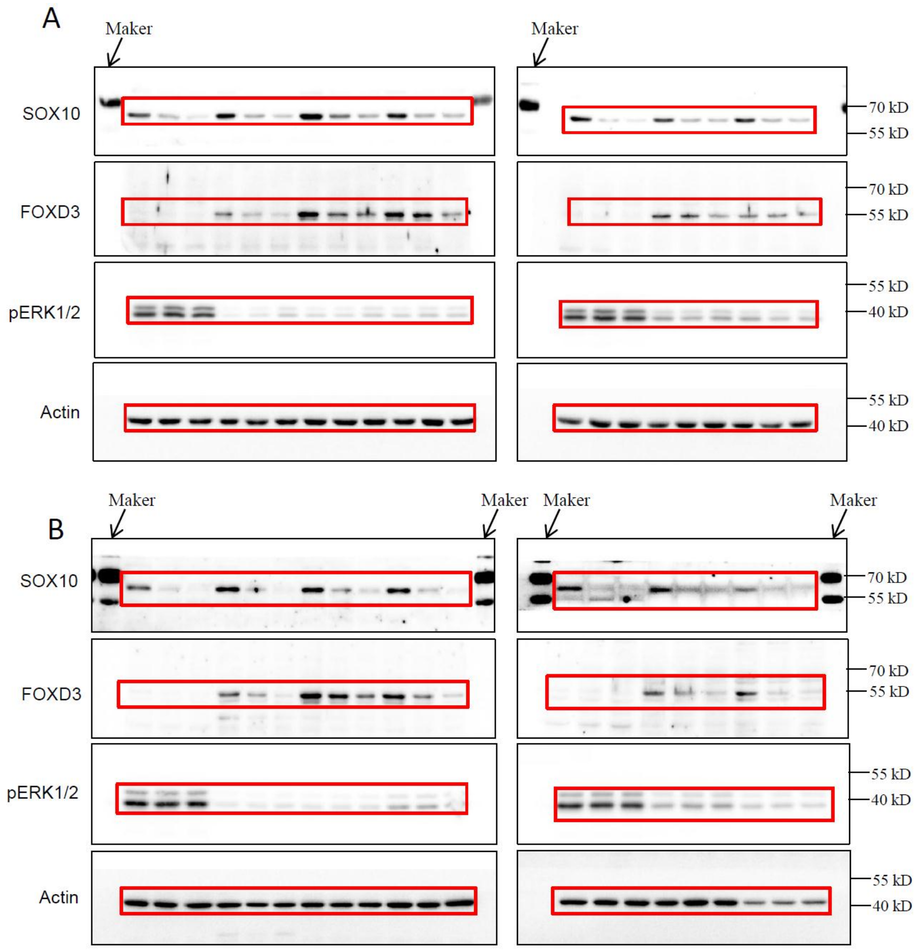

Supplementary Fig. 16. Uncropped western blot images of Supplementary Figure 1a and 1b.

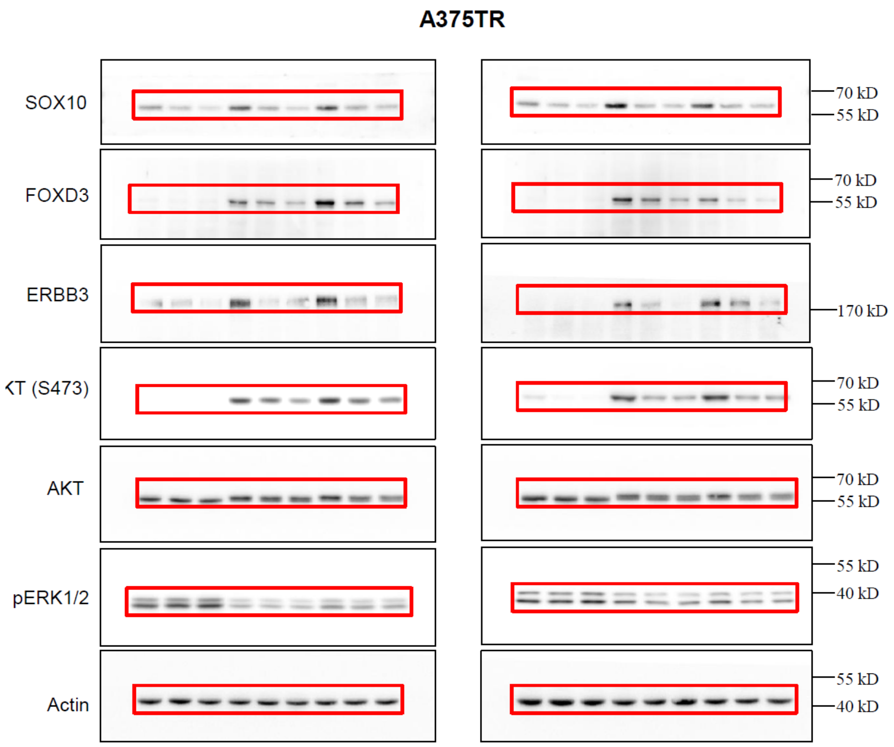

Supplementary Fig. 17. Uncropped western blot images of Supplementary Figure 2-1.

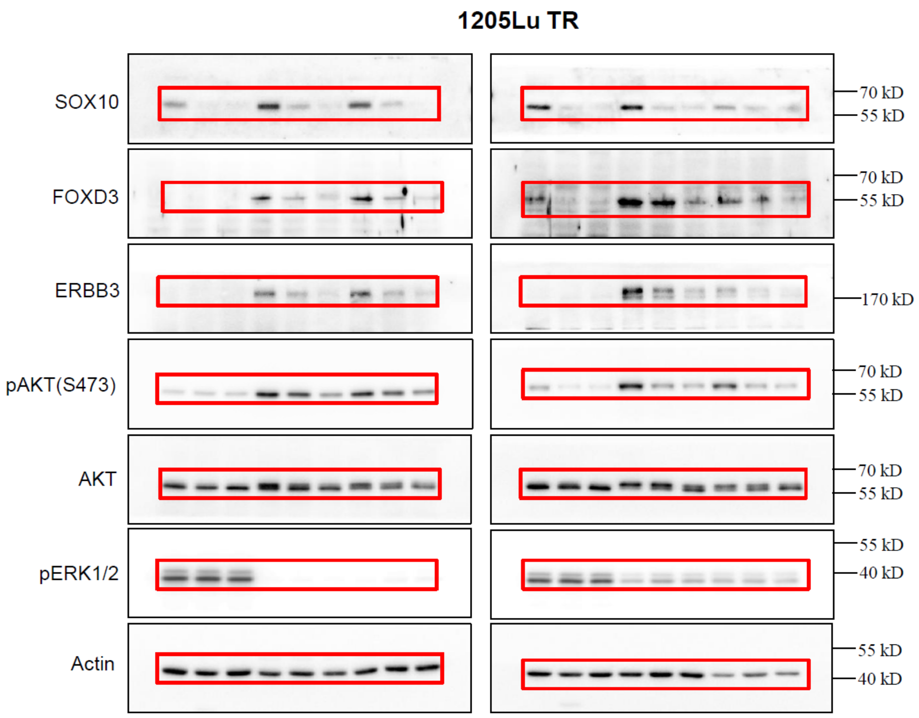

Supplementary Fig. 18. Uncropped western blot images of Supplementary Figure 2-2.

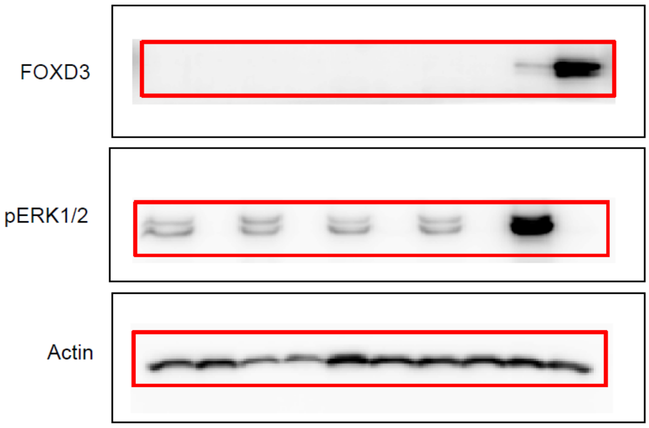

Supplementary Fig. 19. Uncropped western blot images of Supplementary Figure 3.
